# Comprehensive analysis of liver and blood miRNA in precancerous conditions

**DOI:** 10.1038/s41598-020-78500-1

**Published:** 2020-12-10

**Authors:** Tomohiro Umezu, Koichi Tsuneyama, Kohsuke Kanekura, Michiyo Hayakawa, Toshihito Tanahashi, Mitsuoki Kawano, Y-h Taguchi, Hidenori Toyoda, Akihiro Tamori, Masahiko Kuroda, Yoshiki Murakami

**Affiliations:** 1 Department of Molecular Pathology, Tokyo Medical University, Shinjuku 6-1-1, Shinjuku-ku, Tokyo, 160-8402 Japan; 2 Department of Pathology and Laboratory Medicine, Institute of Biomedical Sciences, Tokushima University Graduate School, Tokushima, 770-8503 Japan; 3 Department of Pathology and Cell Regulation, Kyoto Prefectural University of Medicine, Kyoto, 602-0841 Japan; 4 Tokushima Prefecture Naruto Hospital, Naruto, 772-8503 Japan; 5 Department of Human Nutrition, Faculty of Contemporary Life Science, Chugokugakuen University, Okayama, 701-0197 Japan; 6 Department of Physics, Chuo University, Tokyo, 112-8551 Japan; 7 Department of Gastroenterology, Ogaki Municipal Hospital, Ogaki, 503-8502 Japan; 8 Department of Hepatology, Graduate School of Medicine, Osaka City University, Osaka, 545-8585 Japan

**Keywords:** Hepatocellular carcinoma, miRNAs

## Abstract

Streptozotocin administration to mice (STZ-mice) induces type I diabetes and hepatocellular carcinoma (HCC). We attempted to elucidate the carcinogenic mechanism and the miRNA expression status in the liver and blood during the precancerous state. Serum and liver tissues were collected from STZ-mice and non-treated mice (CTL-mice) at 6, 10, and 12 W. The exosome enriched fraction extracted from serum was used. Hepatic histological examination and hepatic and exosomal miRNA expression analysis were serially performed using next-generation sequencing (NGS). Human miRNA expression analysis of chronic hepatitis liver tissue and exosomes, which were collected before starting the antiviral treatment, were also performed. No inflammation or fibrosis was found in the liver of CTL-mice during the observation period. In STZ-mice, regeneration and inflammation of hepatocytes was found at 6 W and nodules of atypical hepatocytes were found at 10 and 12 W. In the liver tissue, during 6–12 W, the expression levels of let-7f-5p, miR-143-3p, 148a-3p, 191-5p, 192-5p, 21a-5p, 22-3p, 26a-5p, and 92a-3p was significantly increased in STZ-mice, and anti-oncogenes of their target gene candidates were down-regulated. miR-122-5p was also significantly down-regulated in STZ-mice. Fifteen exosomal miRNAs were upregulated in STZ-mice. Six miRNAs (let-7f-5p, miR-10b-5p, 143-3p, 191-5p, 21a-5p, and 26a-5p) were upregulated, similarly to human HCC cases. From the precancerous state, aberrant expression of hepatic miRNAs has already occurred, and then, it can promote carcinogenesis. In exosomes, the expression pattern of common miRNAs between mice and humans before carcinogenesis was observed and can be expected to be developed as a cancer predictive marker.

## Introduction

Hepatocellular carcinoma (HCC) represents about 90% of primary liver cancers and constitutes a major global health problem. HCC incidence increases progressively with an advanced age in all populations, reaching a peak at 70 years^1,2^. Approximately 90% of HCCs are associated with chronic viral hepatitis and alcoholism. Recently, HCC occurrence in non-alcoholic steatohepatitis (NASH)/metabolic syndrome patients has gradually increased^3^.

There are several known animal models for analysis of hepatocarcinogenesis. In the diethylnitrosamine-induced model, HCC occurs at an average of 50 weeks (W)^4–6^, and while the Solt-Farber method requires surgical invasion, HCC occurs at 4–8 W^7,8^. Newborn male *ddY, Institute for Animal Reproduction* (DIAR)-nSTZ mice we adopted have had an HCC onset at 12–19 W, and the process of HCC development from dysplastic nodule to well-differentiated HCC involved the expression of tumor markers (Glypican-3 and heat shock protein 70)^9^. STZ is a glucosamine-nitrosourea compound, which is taken up into cells only via glucose transporter GLUT2. The toxicity to pancreatic β cells is derived from their high expression of GLUT2^10–12^. As a result, type I diabetes is induced in mice by high dose STZ or immediately after birth.

It has been reported that Diabetes Mellitus (DM) and insulin resistance are epidemiologically established risk factors for HCC^13–15^. However, the exact biological mechanism underlying the link between diabetes mellitus (DM) and HCC is not completely understood.

Hyperinsulinemia increases the secretion of matrix proteins and other hepatic fibrosis precursor cells by hepatic stellate cells^16^, decreases mitochondrial fatty acid γ-oxidation^17^, hepatocyte injury, inflammation, liver-related fibrosis. Moreover, hyperglycemia and insulin are key-factors in the progression of fibrosis in patients with NASH through the up-regulation of connective tissue growth factor. Importantly, insulin resistance is independently associated with the progression of liver fibrosis, another risk factor of HCC^16^.

Alpha Fetoprotein (AFP) is the most commonly used HCC biomarker, but it lacks sensitivity and specificity in detecting early HCC^18^. In addition, up to 40–50% of HCCs do not produce AFP; thus, there is a limit to using AFP alone for HCC detection. Analysis of cohort studies showed that the sensitivity of AFP for detecting early HCC ranged from 39 to 65% and its specificity ranged from 76 to 97%^19,20^. miRNAs are small non-coding RNAs that play important regulatory roles in various processes, such as cell development, differentiation, and proliferation^21^. Attempts have been made to diagnose liver diseases using miRNA in blood. Serum miRNA-21 levels have been shown to be elevated in HCC patients and to also distinguish between a cirrhotic status and an HCC tumor stage^22,23^. It has been reported that miRNA expression patterns in exosomes are associated with the stage of liver fibrosis and the degree of liver inflammation^24^.

In this study, we serially observed hepatocarcinogenesis model mice without developing liver fibrosis, and then analyzed the carcinogenic mechanism based on miRNA expression analysis in the liver tissue before carcinogenesis and attempted to develop a cancer prediction method using miRNA from the exosome-rich fraction.

## Results

### Pathological findings during hepatocarcinogenesis

**Figure 1:**
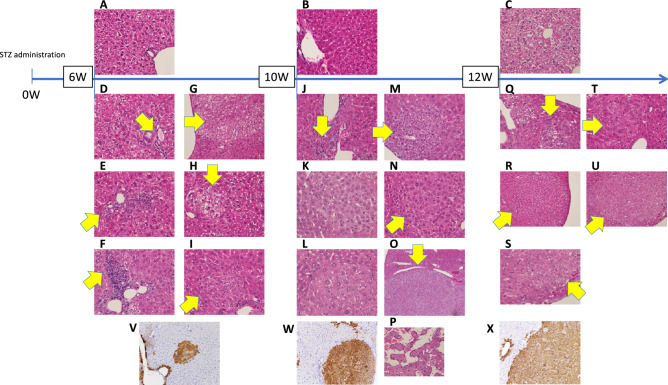
Changes in histopathology with the course of hepatocarcinogenesis. Histopathological images were evaluated by HE staining and glutamine synthetase (GS) immunostaining. Normal hepatic histology without any inflammatory changes in CTL-mice at 6 W (**A**), 10 W (**B**), and 12 W (**C**). STZ-mice (6 W) (**D**–**I**, **V**): Hepatocyte degeneration is seen mildly in the background (**D**–**I**), and mild portal infiltration of lymphocytes (**D**, **F**) and bile ductular hyperplasia (**E**) are observed. Small clusters of atypical hepatocytes showing mild nuclear atypia with hydropic cytoplasmic change (< 1 mm in size) were observed (**G**–**I**). GS is diffusely positive in these atypical hepatocytes (**V**). STZ-mice (10 W) (**J**–**P**, **W**): Hepatocyte degeneration is mildly observed in the background (**J**–**L**). Infiltration of lymphocytes and hyperplasia of bile ductules are scatteredly observed (**J**–**L**). Aypical hepatocellular nodules (1–6 mm in size) (**M**–**O**) those resembling low–high grade dysplastic nodules and HCC showing thin trabecular pattern with cellular atypia (**P**) are observed. GS is diffusely positive in these atypical hepatocellular nodules and HCC (**W**). STZ-mice (12 W) (**Q**–**U**, **X**): In the background liver, degeneration of hepatocytes, infiltration of lymphocytes, and hyperplasia of bile ductules were slightly unnoticeable (**Q**–**S**). Aypical hepatocellular nodules (1–6 mm in size) (**R**-) as well as small clusters of atypical hepatocytes (< 1 mm) (**Q**) are mixedly observed. Several atypical hepatocellular nodules showing invasive growth (**S**) mimics those found in human well-differentiated HCC (**X**). The part showing each feature is indicated by a yellow arrow.

We sacrificed STZ-treated mice (STZ-mice) and control mice (CLT-mice) at 6, 10, and 12 W. In CTL-mice, no inflammation or fibrosis was found in the liver during the observation period. On the other hand, degeneration of hepatocytes, infiltration of lymphocytes, the proliferation of bile ductules, and small clusters of atypical hepatocytes (< 1 mm in size) were observed at 6 W in STZ-mice. In addition to these changes, atypical hepatocellular nodules (1–6 mm in size) resembling low–high grade dysplastic nodules were also seen at 10 W in STZ-mice. Moreover, HCC showing a thin trabecular pattern with invasive growth was observed in one case. Atypical hepatocellular nodules (1–6 mm in size) were still observed; however, degeneration of hepatocytes, infiltration of lymphocytes, and proliferation of bile ductules became inconspicuous at 12 W in STZ-mouse (Fig. 1). Several atypical hepatocellular nodules (4–6 mm) at 12 W mimics those found in human well-differentiated HCC, which showed invasive growth without fibrous capsules. From small clusters of atypical cells at 6 W to large atypical hepatocellular nodules at12W, glutamine synthetase (GS), which is a marker of HCC were consistently positive (Fig. 1).

### Evaluation of precancerous status using comprehensive gene expression

Using all the information regarding liver and exosome miRNA, it was examined whether STZ-mice and CLT-mice could be discriminated using gene expression analysis to differentiate between the precancerous and the normal states. Information on 15 miRNAs (let-7f-5p, miR-10a-5p, miR-10b-5p, miR-122-5p, miR-143-3p, miR-148a-3p, miR-191-5p, miR-192 -5p, miR-21a-5p, miR-22-3p, miR-26a-5p, miR-30a-5p, miR-486a-5p, miR-486b-5p, and miR-92a-3p) in the liver and in exosomes and on 95 mRNAs in the liver (Supplementary Table 1, Fig. 3, and Supplementary Fig. 1) was chosen using tensor decomposition and this gene expression information was able to differentiate both groups with a 100% accuracy (Table 1).

**Table 1:** Discrimination between normal and precancerous states using comprehensive genetic analysis.

Result	Prediction	
	Normal	Pre-cancer state
Normal	9	0
Pre-cancer state	0	9

### Gene expression analysis in the liver

**Figure 2:**
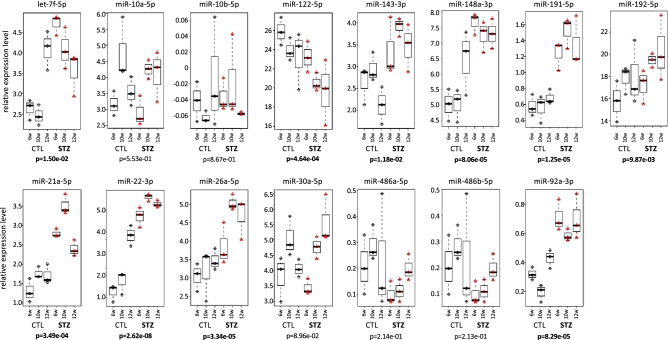
Expression pattern of miRNA in the liver. The expression level of 15 miRNAs in the liver from serial collection (6 W, 10 W, and 12 W) was shown in STZ-mice and in CTL-mice. Statistical analysis was performed using the student-t test, and a difference was considered statistically significant for a *p* < 0.05 (bold letter). The vertical axis represents the expression level of miRNA.

**Figure 3:**
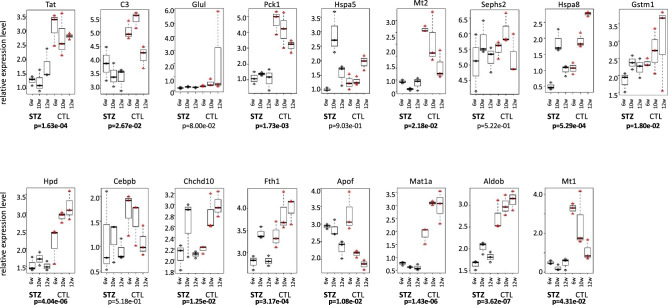
Expression pattern of target genes candidates of miRNAs with different expression in STZ-mice and in CTL-mice. The expression of 17 genes from the liver samples that were serially collected is shown. Statistical analysis was performed using the student-t test, and a difference was considered statistically significant for a *p* < 0.05 (bold letter). The vertical axis represents the expression level of miRNA.

Ten miRNAs of 15 selected hepatic miRNAs showed significant differences in expression in both STZ- and CTL-mice. Among them, only miR-122-5p had a high expression in CTL-mice, and the others had a high expression in STZ-mice (Fig. 2). The expression of miR-122 was suppressed in STZ-mice, compared to CTL-mice, and the expression of both groups decreased gradually from 6 to 12 W. The expression of miR-143-3p, 148a-3p, 191-5p, 192-5p, 21a-5p, 22-3p, 26a-5p, and 92a-3p was higher in STZ-mice than in CTL-mice and tended to increase gradually from 6 to 12 W. The expression of let-7f-5p was higher in STZ-mice than in CTL-mice, and its expression pattern was opposite at 12 W. The expression of miR-30a-5p was higher in STZ-mice than in CTL-mice, and the expression pattern was opposite at 6 and 10 W. Then, the target gene candidates of 15 hepatic miRNAs were investigated from the 95 mRNAs. The selection of the target genes was first narrowed down by using tarbase ver. 7 (http://diana.imis.athena-innovation.gr/DianaTools/index.php?r=tarbase/index) (Table 2, Fig. 3, and Supplementary Fig. 1).

**Table 2:** Summary of miRNA target genes candidates.

Gene ID	Gene	miRNA1	miRNA2	miRNA3	Function	Match	PMID
ENSMUSG 00000049091	Sephs2	let-7f-5p	miR-22-3p		Anti-cancer	○	30469315
ENSMUSG 00000001670	Tat	let-7f-5p			Anti-cancer	○	20209601
ENSMUSG 00000029445	Hpd	miR-143-3p			NI		
ENSMUSG 00000037798	Mat1a	miR-22-3p			Anti-cancer	○	27981602
ENSMUSG 00000056501	Cebpb	miR-22-3p	miR-92a-3p		Cancer	×	30808546
ENSMUSG 00000049422	Chchd10	miR-22-3p			NI		
ENSMUSG 00000024661	Fth1	miR-21a-5p	miR-26a-5p		Anti-cancer	○	32476555
ENSMUSG 00000026473	Glul	miR-22-3p	miR-26a-5p	miR-30a-5p	Cancer	×	31986891
ENSMUSG 00000015656	Hspa8	miR-21a-5p			Cancer	×	29516568
ENSMUSG 00000058135	Gstm1	miR-26a-5p			Anti-cancer	○	26045716
ENSMUSG 00000026864	Hspa5	miR-26a-5p	miR-30a-5p		Anti-cancer	○	29260979
ENSMUSG 00000031762	Mt2	miR-26a-5p			Anti-cancer	○	25871729
ENSMUSG 00000027513	Pck1	miR-26a-5p			Anti-cancer	○	29335519
ENSMUSG 00000028307	Aldob	miR-92a-3p			Anti-cancer	○	30760861
ENSMUSG 00000024164	C3	let-7f-5p			Cancer	×	21953030
ENSMUSG 00000031765	Mt1	miR-30a-5p			Anti-cancer	○	29873415
ENSMUSG 00000047631	Apof	miR-122-5p			NI		

miRNA: single or multiple miRNAs that recognize genes; function: with or without carcinogenic potential; NI (no information) shows no information on carcinogenic potential; match: ○ is gene expression pattern and carcinogenic potential match; PMID: citations regarding carcinogenesis are given.

Furthermore, enrichment analysis revealed that many of the miRNA target genes candidates are involved in the pathways of type I diabetes and hepatocellular carcinoma (http://amp.pharm.mssm.edu/Enrichr/enrich?dataset=f87d36a7f37eb102536c11c69bd3e933) (Table 3).

**Table 3:** Summary of enrichment analysis.

Term	Gene expression	Overlap	Adjusted *p* value	Odds Ratio	Combined score		
GSE2127	Down	25/298	7.52E−25	21.79	1322.02		
	Up	19/302	3.45E−16	16.34	657.83		
GSE4612	Down	21/237	3.35E−21	23.01	1192.10		
	Up	22/363	1.51E−18	15.74	723.78		
GSE11	Down	4/321	1.07E−01	3.24	10.82		
	Up	2/279	4.89E−01	1.86	2.29		
GSE1659	Down	1/248	8.88E−01	1.05	0.50		
GSE2254	Up	6/352	1.07E−02	4.43	26.77		
	Down	3/200	1.18E−01	3.90	12.35		
	Up	8/400	1.12E−03	5.19	45.72		
GSE4616	Down	5/343	3.97E−02	3.79	17.29		
	Up	6/257	2.79E−03	6.06	46.47		

Expression pattern	Term	Related genes					
Down	GSE2127	C3	Vtn	Serpina1C	Cyp2d9	Cyp2f2	Car3
		Fgb	Fga	Gpx1	Mup10	Fgg	Apoa2
		Serpina3k	Chchd10	Mup7	Mat1a	Apoa5	Cyp8b1
		Mup3	Gnmt	Fabp1	Rbp4	Aldob	Cyp2e1
		Hpd					
Up		Hspa8	Gstm1	Ahsg	Gstp1	Hp	Apoa1
		Apoa4	Mt2	Mt1	Sult1a1	Hpx	Ftl1
		Lrg1	Cyp2a5	Fth1	Alb	Thrsp	Scd1
		Glul					
Down	GSE4612	Car3	Mup10	Hspa5	Fgg	Apoa2	Serpina3k
		Apoa1	Chchd10	Apoc3	Mat1a	Cyp8b1	Gnmt
		Fabp1	Rbp4	Serpina1c	Cyp2d9	Ttr	Thrsp
		Cyp2e1	Hpd	Glul			
Up		Fga	Gpx1	Gstm1	Ahsg	Hp	Apoa4
		Mup7	Apoa5	C3	Sult1a1	Vtn	Hpx
		Ftl1	Lrg1	Cyp2a5	Fth1	Apof	Aldob
		Scd1	Apoe	Cyp2f2	Pck1		
Down	GSE11	Hpx	Tat	Apoa4	Sepp1		
Up		Vtn	Gpx1				
Down	GSE1659	Chchd10					
Up		Car3	Gstm1	Fth1	Mt2	Mt1	Eef2
Down	GSE2254	Fabp1	Apoa1	Apoa4			
Up		C3	Hspa8	Hspa5	Fth1	Hp	Mt1
		Apoe	Eef2				
Down	GSE461	Hspa8	Gstp1	Chchd10	Thrsp	Scd1	
Up		Gstm1	Lrg1	Fth1	Mt2	Mt1	Eef2

A list of phenotypes obtained by enrichment analysis and genes related to phenotype is shown. GSE2127; carcinoma, hepatocellular C0019204 mouse GSE2127 sample 300, GSE4612; carcinoma, hepatocellular C0019204 mouse GSE4612 sample 315, GSE11; type 1 diabetes mellitus C0011854 mouse GSE11 sample 173, GSE1659; type 1 diabetes mellitus C0011854 mouse GSE1659 sample 346, GSE2254; type 1 diabetes mellitus C0011854 mouse GSE2254 sample 204, GSE4616; type 1 diabetes mellitus C0011854 mouse GSE4616 sample 363 The genes with underline are shown to be in agreement with the expression pattern.

Enrichment analysis was performed to clarify the significance of the selected 95 genes. As a result, many common genes were found in the two mouse HCC models (GSE2127 and GSE4612) and four types of type I diabetic mouse models (GSE11, GSE1659, GSE2254, and GSE4616) (Table 3).

### miRNA expression analysis in the exosome rich fraction

**Figure 4:**
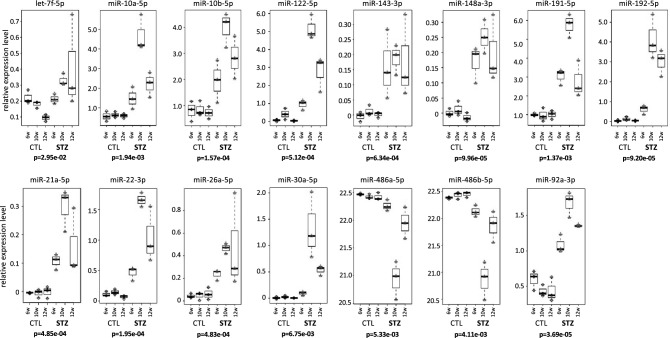
Expression pattern of miRNA in the exosome rich fraction. The expression level of 15 miRNAs from serial collection (6, 10, and 12 W) is shown for STZ-mice and CTL-mice. Statistical analysis was performed using the student-t test, and a difference was considered statistically significant for a *p* < 0.05 (bold letter). The vertical axis represents the expression level of miRNA.

The expression level of the 13 miRNAs (let-7f-5p, miR-10a-5p, 10b-5p, 122-5p, 143-3p, 148a-3p, 191-5p, 192-5p, 21a-5p, 22-3p, 26a-5p, 30a-5p, and 92a-3p) in STZ-mice was significantly higher than that in CTL-mice. The expression level of miR-486a-5p and 486b-5p in CTL-mice was significantly higher than that in STZ-mice (Fig. 4).

### Comparison of miRNA expression pattern between human clinical specimens and mouse carcinogenic model

**Figure 5:**
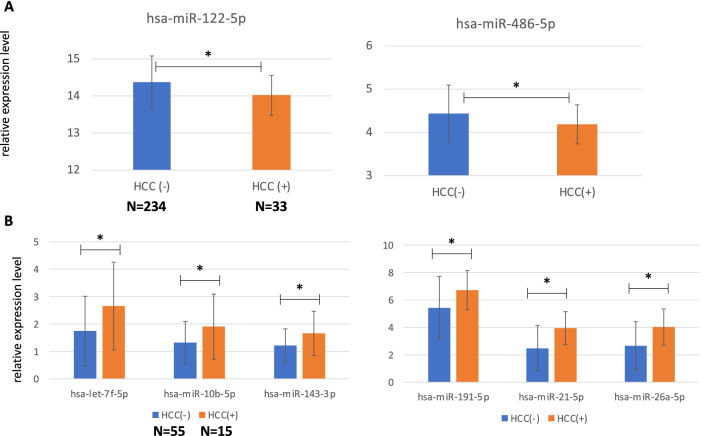
Analysis of miRNA expression in specimens collected before the antiviral treatment. Expression of hsa-miR-122-5p and 486-5p in the liver tissue (**A**) and expression analysis of hsa-let-7f-5p, miR-10b-5p, miR-143-3p, miR-191-5p, miR-21-5p, and miR-26a-5p in the exosome rich fraction (**B**) are shown, respectively. Statistical analysis was performed using the student-t test, and a difference was considered statistically significant for a *p* < 0.05.

To examine the significance of the gene expression analysis results obtained in the precancerous state of mice, analysis was performed using human chronic hepatitis specimens that had been subjected to long-term observation. Human miRNA expression from the liver tissue of 267 chronic hepatis C patients was analyzed by using microarrays (Supplementary Table 2). Liver tissues were collected via needle biopsy before starting the anti-viral treatment. All patients were confirmed as not having HCC by using tumor markers and imaging analysis at the time of sample collection. During the follow-up, after treatment, 33 of 267 patients developed HCC. Comparing the hepatic miRNA expression pattern between the HCC and the no-HCC groups, the expression level of miR-122-5p and 486-5p in the no-HCC group was significantly lower than that in the HCC group (Fig. 5A).

The serum of 70 chronic hepatitis C and liver cirrhosis type C patients from another cohort of a liver tissue study was collected before the antiviral treatment (Supplementary Table 3). The exosome rich fraction was collected from the serum, as in the mouse experiment. Fifteen of 70 patients developed HCC during the follow-up period. The expression level of 6 miRNAs (let-7f-5p, miR-10b-5p, 143-3p, 191-5p, 21-5p, and 26a-5p) in the HCC group was significantly higher than that in the no-HCC group and the expression pattern of these 6 miRNAs was in agreement with the mouse model (Fig. 5B).

## Discussion

No liver fibrosis or liver inflammation was observed during hepatocarcinogenesis in the DIAR mice with STZ treatment^9^; therefore, the abnormal gene expression obtained in this analysis was less affected by liver fibrosis or inflammation.

Regarding the effect of miRNA expression on STZ administration, it was only reported that miR-1302 is regulated by glucokinase at the onset of diabetes^25^. The expression of 7 miRNAs in liver tissues was enhanced by STZ administration. Importantly, HCC had not yet developed when miRNA expression changed. Seven of the up-regulated miRNAs had 15 target genes candidates, 9 of which were tumor suppressor genes. It has been reported that 3 miRNAs (miR-191-5p, 21-5p, and 92a-3p)^26–28^ out of 7 miRNAs have a carcinogenic potential. On the other hand, miR-122-5p has been reported to be involved in many liver metabolisms^29,30^ and its expression suppression is associated with a decreased liver function and carcinogenesis^31,32^.

**Figure 6:**
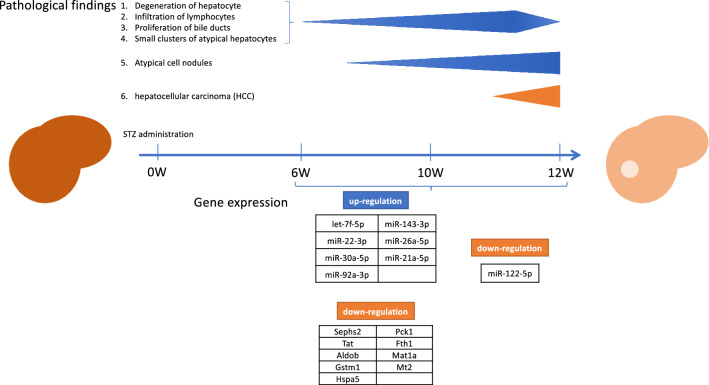
Summary of changes in histopathology and changes in gene expression in liver tissue seen during carcinogenesis. The upper row shows the histological changes in the liver observed from 6 to 12 W. The lower row illustrated changes in hepatic gene expression induced by STZ administration. The expression patterns of miRNA and mRNA are shown with reference to the CTL observed when there is a change in histopathological condition.

Importantly, the down-regulation of miR-122-5p and the up-regulation of miR-191-5p, 21-5p, and 92a-3p in the liver tissue has already occurred, even in the precancerous state. According to enrichment analysis, the genes used for classification were the commonly involved in type I diabetes and liver carcinogenesis pathways. Taken together, an aberrant expression pattern of genes observed in the liver of STZ-mice is associated with a high carcinogenic potential (Fig. 6 and Table 2).

It is well known that exosomes contain miRNA, among others, and carry out cell-to-cell communication^33^. Although the recovery of exosomes is less invasive than the collection of tissues, it is attracting attention as a liquid biopsy because much information can be obtained from it^34^. Although the standard recovery of exosomes involves an ultracentrifugation method, it takes time and effort; thus, this time, in order to use a simple and reliable method, exosomes were collected using aggregation with polyethylene glycol. This method also aggregates the structures that are similar to exosomes; thus, we analyzed miRNAs in exosome-rich fractions, instead of miRNAs recovered only from exosomes^35^. There was no significant correlation between miRNA expression in the liver tissue and in exosomes, but all 15 selected miRNAs showed a significant difference in expression between STZ-mice and CTL-mice. The reason why the difference of miRNA profiles between STZ-mice and CTL-mice were not reflected in exosome-rich fraction remains unknown. The changes of miRNA expression level in the cells facing hepatic vein/artery which have more chance to leak miRNA into circulatory system might be over-represented in the exosome-rich fraction. Further studies such as in situ hybridization will help us to clarify it.

Four up-regulated miRNAs (miR-10b-5p, miR-21-5p, miR-122-5p, and miR-148a-3p), have been reported as early HCC diagnostic markers^36–38^; however, the abnormal expression of these miRNAs occurred in a cancer state, and not in a precancerous state. A comparison of human specimens before starting the antiviral therapy in chronic hepatitis and cirrhotic conditions revealed that the expression of 6 miRNAs (let-7f-5p, miR-10b-5p, 143-3p, 191-5p, 21-5p, and 26a-5p) in the exosomes was higher in patients who developed cancer after the end of treatment than in those who did not. Human samples were observed to have HCV infection and liver fibrosis, whereas mouse specimens had neither viral infection nor liver fibrosis. However, the expression pattern of 6 miRNAs in exosomes related to carcinogenesis were the same between humans and mice. This result is considered interesting from the point of view of carcinogenesis. Recently, there has been a comprehensive report on circulating mRNA analysis, and among the mRNAs whose expression is different between HCC and liver cirrhosis and the mRNA in liver tissue used for this analysis, Alb, ApoA1, ApoA2, and Ftl was common with both study.

Although there are differences in the analysis species between human clinical specimens and mouse experimental models, it is considered to be an interesting result for elucidating the mechanism of hepatocarcinogenesis^39^.

In conclusion, a comprehensive analysis of gene expression in the liver tissue showed that a down-regulation of tumor suppressor genes and an up-regulation of oncogenes was related to the development of the precancerous status to HCC. Furthermore, analysis of miRNAs in exosomes is expected not only to be a diagnostic marker for early HCC, but also to be a predictive marker for carcinogenesis in precancerous conditions.

## Materials and methods

### Animal models

We used a DIAR mouse model. Details on breeding methods and procedures were described previously^9^. In brief, newborn male DIAR mice were prepared at the Institute of Animal Reproduction (Ibaraki, Japan). These mice were divided into two groups based on the STZ treatment. At 1.5 days after birth, STZ was subcutaneously injected (60 mg/kg) into the treated group (STZ-mice), whereas the same volume of physiologic solution was injected into the control group (CTL-mice). The STZ and CTL-groups were comprised of 9 mice each. All mice were maintained on a regular diet. Mice in each group were physiologically and histopathologically assessed at 6, 10, and 12 W of age. All institutional and national guidelines for the care and use of laboratory animals were followed. This study was performed in accordance with the animal experiment guidelines specified by the Institute for Animal Reproduction (Ibaraki, Japan), which strictly observed the rules of guidance on animal research ethics from the International Association of Veterinary Editors’ Consensus Author Guidelines on Animal Ethics and Welfare.

### Histological evaluation

The procedure of mouse liver tissue specimen was described previously. Briefly tissue sample was fixed in 10% neutral buffered formalin formaldehyde, embedded in paraffin, and cut into 4-μm-thick sections. Deparaffinized sections were stained with hematoxylin–eosin dehydrated in 100% ethanol, washed excess pigment with xylene, mounted with NEW M-X (Matsunami Glass Industries, Osaka, Japan)^40^.

The procedure of immunostaining is described previously^41^. Briefly, rabbit polyclonal anti-glutamine synthetase (GS) antibody (ab49873, abcam, Cambridge, UK) antibody was used for HCC diagnosis. After deparaffinization, the sample was heated with an antigen recovery solution. Subsequently, 5% H_2_O_2_ in methanol was treated to block endogenous peroxidase for 5 min. After incubating with 5% bovine serum albumin (Sigma-Aldrich Japan K.K., Tokyo, Japan) to block non-specific binding, the specimens were incubated overnight at 4° C in prediluted primary antibody. Immune-staining was visualized by using 3,3′-Diaminobenzidine (SK4100; Vector Laboratories Inc., Burlingame, CA, USA)—EnVision Polymer—horseradish peroxidase (K4001; Dako Denmark A/S, Glostrup, Denmark). Sections were lightly counterstained using hematoxylin to make the image clearer.

### RNA extraction

The exosome-rich fraction was collected from 900 μL of serum by using ExoQuick (System Biosciences, Palo Alto, CA, USA). Total RNA was extracted from the tissue samples and exosome-rich fractions using an RNeasy Mini Kit (Qiagen, Hilden, Germany). The concentration, integrity number, 28S/18S ratio, and the sample size of the extracted RNA were qualified using an Agilent 2100 Bioanalyzer (Agilent Technologies, Santa Clara, CA, USA).

### Next-generation sequencing analysis for miRNA

For establishment of cDNA library, total RNA was fractionated into 18–30 nt small RNA on a 6% polyacrylamide gel, and then PCR was performed after an adapter sequence was added using TruSeq Small RNA Library Prep Kit (Illumina, San Diego, CA, USA). Detailed procedure have been shown previously^42^. The cDNA library was sequenced using the Illumina MiSeq system (Illumina, San Diego, CA, USA). All sequence data is stored on NCBI's Gene Expression Omnibus and can be accessed via GEO accession number GSE153581.

Raw small RNA sequencing data were analyzed using CAP-miRSeq, integrated analysis tools, to derive miRNA expression from the fastq files. CAP-miRSeq integrates several individual tools that process fastq files, such as cutadupt, fastqc, and miRDeep2. Using CAP-miRSeq, the generated fastq files are converted to miRNA expression profiles (miRBase 21 is used as a reference). Finally, the generated mature_miRNA_expression.xls file was used as an miRNA expression profile.

### Next generating sequencing analysis

Beijing Genomics Institute BGI (Hong Kong, China) performed the preparation of cDNA library and sequencing. A brief description of cDNA library synthesis is as follows. After extraction of total RNA, DNase I treatment was performed to remove DNA contamination, and mRNA was extracted using magnetic beads having oligo dT fragments. cDNA is synthesized after mRNA fragmentation. After nucleic acid purification, add adenine and add adapter sequence. Quality and quantity of cDNA library was checked using the Agilent 2100 Bioanaylzer and StepOnePlus Real-Time PCR System (Thermo Fisher Scientific Inc. Waltham MA), and Illumina HiSeqTM2500 was used for the sequence^43^.

All sequence data is stored in NCBI's Gene Expression Omnibus and can be accessed via GEO accession number GSE153580.

### Human samples

Serum was obtained from 70 patients, 12 W after achieving a sustained viral response using treatment with a direct anti-viral agent (Supplementary Table 1), and chronic hepatitis (CH) C tissue samples were collected via needle biopsy before antiviral treatment in all 267 cases (Supplementary Table 2). This study was conducted according to the guidelines of the 1975 Declaration of Helsinki (2013 version). Written informed consent was obtained from all patients prior to treatment. The study protocol was approved by the Ethics Committees of Osaka City University Hospital (No. 1358) and Ogaki Municipal Hospital (No. G-219), respectively.

### Microarray analysis for miRNA

One hundred nanograms of total RNA from tissue samples and 60 ng of total RNA of the exosome rich fractionated serum were analyzed using 3D-Gene miRNA microarray (Toray Industries, Inc., Kanagawa, Japan). Comprehensive miRNA expression analysis was performed using a 3D-Gene miRNA Labeling Kit and a 3D-Gene Human miRNA Oligo Chip (Toray Industries, Inc.), both of which could detect 2,555 miRNA sequences in miRBase release 20 (http://www.mirbase.org/). All microarray datasets from this study were in conformance with the “Minimum Information About a Microarray Experiment” guidelines and are publicly available in the GEO database (GSE147892 for liver tissues and GSE119159 for exosomes).

### Statistical analysis

Suppose we have three liver mRNA/miRNA expression profiles, ${x}_{ij}^{mRNA:liver} \in {\mathbb{R}}^{N\times M}$, where *N* is total number of mRNAs and *M* = 18 is the number of samples, the liver miRNA expression profile is ${x}_{kj}^{miRNA:liver} \in {\mathbb{R}}^{K\times M}$, where *K* is the total number of miRNAs, and the serum miRNA expression profile is ${x}_{kj}^{miRNA:serum}\in {\mathbb{R}}^{N\times M}$. These are converted to tensor, ${x}_{ijkm}\in {\mathbb{R}}^{N\times M\times K\times 2}$, where ${x}_{ijk1}= {x}_{kj}^{miRNA:liver} \times {x}_{ij}^{mRNA:liver}$, and ${x}_{ijk2}= {x}_{kj}^{miRNA:serum} \times {x}_{ij}^{mRNA:liver}.$ ${x}_{ijkm}$ was decomposed using higher order singular value decomposition (HOSVD)^44^ algorithm, as follows:

$${x}_{ijkm}= \sum_{{l}_{1}=1}^{N}\sum_{{l}_{2}=1}^{M}\sum_{{l}_{3}=1}^{K}\sum_{{l}_{4}=1}^{2}G({l}_{1}{l}_{2}{l}_{3}{l}_{4}){u}_{{l}_{1}i}{u}_{{l}_{2}j}{u}_{{l}_{3}k}{u}_{{l}_{4}m}$$

where $G\in {\mathbb{R}}^{N\times 2M\times K\times 2}$ is a core tensor, ${u}_{{l}_{1}i}\in {\mathbb{R}}^{N\times N}$,${u}_{{l}_{2}j}\in {\mathbb{R}}^{M\times M}$,${u}_{{l}_{3}k}\in {\mathbb{R}}^{K\times K}$, and ${u}_{{l}_{4}m}\in {\mathbb{R}}^{2\times 2}$ are singular value matrices that are orthogonal matrices. Eighteen samples are composed of nine STZ samples and nine control samples, each of which are composed of three 6 W samples, three 10 W samples, and three 12 W samples.

In order to select biologically valuable mRNAs and miRNAs, we need to select the ${u}_{{l}_{1}i}$ and ${u}_{{l}_{3}k}$ to be used, respectively. In order to do that, we first must identify which ${u}_{{l}_{2}j}$ are biologically informative, i.e., which ones are distinct between the STZ samples and the control samples, or between time points. After visual inspection, we noticed that ${l}_{2}=2$ is distinct between STZ and the controls and that ${l}_{2}=3, 4$ are distinct between time points. Next, we tried to find which ${u}_{{l}_{1}i}$ and ${u}_{{l}_{3}k}$ are associated with larger absolute values of $G\left({l}_{1}{l}_{2}{l}_{3}{l}_{4}\right), 2\le {l}_{2}\le$ 4. Then, we realized that $1 \le {l}_{1},{l}_{3}\le 4$ satisfy these requirements. Finally, P-values were attributed to mRNAs and miRNAs, by assuming that ${u}_{{l}_{1}i}$ and ${u}_{{l}_{3}k}$ obey to a Gaussian distribution (null hypothesis), by using χ^2^ distribution:

$${P}_{i}={P}_{{\chi }^{2}}\left[>\sum_{{l}_{1}=1}^{4}{\left(\frac{{u}_{{l}_{1}i}}{{\sigma }_{{l}_{1}}}\right)}^{2}\right],{P}_{k}={P}_{{\chi }^{2}}\left[>\sum_{{l}_{3}=1}^{4}{\left(\frac{{u}_{{l}_{3}i}}{{\sigma }_{{l}_{3}}}\right)}^{2}\right]$$

where ${P}_{{\chi }^{2 }}\left[>x\right]$ is the cumulative χ^2^ distribution whose argument is larger than *x* and ${\sigma }_{{l}_{1}}$ and ${\sigma }_{{l}_{3}}$ are the standard deviations. The P-values were corrected by using the Benjamini–Hochberg criterion^44^. Fifteen miRNAs and 95 mRNAs associated with adjusted P-values lower than 0.01 were selected. These procedures were described in detail previously^44^.

## Supplementary information

Supplementary Information.
