# Supplementary material for: Comprehensive analysis of liver and blood miRNA in precancerous conditions
Source: Sci Rep. 2020 Dec 10;10:21766. doi: 10.1038/s41598-020-78500-1 (PMC7728755; doi:10.1038/s41598-020-78500-1)
Supplement: Supplementary file 1 — Supplementary Information. [file 41598_2020_78500_MOESM1_ESM.pdf]

SUPLEMENTARY INFORMATION

Comprehensive analysis of liver and blood miRNA in precancerous conditions

Tomohiro Umezu[1], Koichi Tsuneyama[2], Kohsuke Kanekura[1], Michiyo Hayakawa[3], Toshihito Tanahashi[4], Mitsuoki Kawano[5], Y-h Taguchi[6], Hidenori Toyoda[7], Akihiro Tamori[8], Masahiko Kuroda[1], Yoshiki Murakami[1*]

[1] Department of Molecular Pathology, Tokyo Medical University, Tokyo 160-8402, Japan.

[2] Department of Pathology and Laboratory Medicine, Institute of Biomedical Sciences, Tokushima University Graduate School, Tokushima 770-8503, Japan.

[3] Department of Pathology and Cell Regulation, Kyoto Prefectural University of Medicine, Kyoto 602-0841, Japan.

[4] Tokushima Prefecture Naruto Hospital, Naruto 772-8503, Japan.

[5] Department of Human Nutrition, Faculty of Contemporary Life Science, Chugokugakuen University, Okayama 701-0197, Japan.

[6] Department of Physics, Chuo University, Tokyo 112-8551, Japan.

[7] Department of Gastroenterology, Ogaki Municipal Hospital, Ogaki 503-8502, Japan.

[8] Department of Hepatology, Osaka City University, Osaka 545-8585, Japan.

* Correspondence to: Yoshiki Murakami, Department of Molecular Pathology, Tokyo Medical University, Shinjuku 6-1-1, Shinjuku-ku, Tokyo 160-8402, Japan.
E-mail yoshikim@tokyo-med.ac.jp

Supplementary information

Supplementary Table 1. List of genes used for classification.

Supplementary Table 2. Clinical data of patients with chronic hepatitis C (liver tissue analysis).

Supplementary Table 3. Clinical data of chronic hepatitis C patients (exosome analysis).

Supplementary Figure 1. Expression pattern of mRNA in STZ- and CTL-mice.

Among the genes used to differentiate between STZ and CTL-mice, the expression pattern of 78 genes is shown. These genes are not the target genes of miRNA shown in Figure 3.

Supplementary table 1. List of genes used for classification

Gene ID	Gene symbol	Gene name	Gene ID	Gene symbol	Gene name
ENSMUSG00000001670	Tat	Tyrosine aminotransferase	ENSMUSG00000058135	Gstm1	Glutathione S-transferase, mu 1
ENSMUSG00000002769	Gnmt	Glycine N-methyltransferase	ENSMUSG00000058207	Serpina3k	Serine peptidase inhibitor, clade A, member 3K
ENSMUSG00000002985	Apoe	Apolipoprotein E	ENSMUSG00000058216	Gstp3	Glutathione S-transferase pi 3
ENSMUSG00000005547	Cyp2a5	Cytochrome P450, family 2, subfamily a, polypeptide 5	ENSMUSG00000058492	NA (NA1)	Sterol carrier protein 2, pseudogene 2
ENSMUSG00000005681	Apoa2	Apolipoprotein A-II	ENSMUSG00000060803	Gstp1	Glutathione S-transferase, pi 1
ENSMUSG00000015656	Hspa8	Heat shock protein 8	ENSMUSG00000061808	Ttr	Transthyretin
ENSMUSG00000017344	Vtn	Vitronectin	ENSMUSG00000063856	gpx1	Glutathione peroxidase 1
ENSMUSG00000022868	Ahsg	Alpha-2-HS-glycoprotein	ENSMUSG00000064341	Nd1 (mt-Nd1)	Mitochondrially encoded NADH dehydrogenase 1
ENSMUSG00000024164	C3	Complement component 3	ENSMUSG00000064345	Nd2 (mt-Nd2)	Mitochondrially encoded NADH dehydrogenase 2
ENSMUSG00000024661	Fth1	Ferritin heavy polypeptide 1	ENSMUSG00000064351	Cox1 (mt-Cox1)	Mitochondrially encoded cytochrome c oxidase I
ENSMUSG00000025479	Rbp4	Cytochrome P450, family 2, subfamily e, polypeptide 1	ENSMUSG00000064352	NA(NA2)	Mitochondrially encoded tRNA serine 1
ENSMUSG00000026473	Cyp2e1	Glutamate-ammonia ligase	ENSMUSG00000064363	Nd4 (mt-Nd4)	Mitochondrially encoded NADH dehydrogenase 4
ENSMUSG00000026864	Glul	Heat shock protein 5	ENSMUSG00000064367	Nd5 (mt-Nd5)	Mitochondrially encoded NADH dehydrogenase 5
ENSMUSG00000027513	Hspa5	Phosphoenolpyruvate carboxykinase 1	ENSMUSG00000064368	Nd6 (mt-Nd6)	Mitochondrially encoded NADH dehydrogenase 6
ENSMUSG00000027559	Pck1	Carbonic anhydrase 3	ENSMUSG00000064370	Cytb	Mitochondrially encoded cytochrome b
ENSMUSG00000028001	Car3	Fibrinogen alpha chain	ENSMUSG00000064372	NA(NA3)	Mitochondrially encoded tRNA proline
ENSMUSG00000028307	Fga	Aldolase B, fructose-bisphosphate	ENSMUSG00000064373	Sepp1	Selenoprotein P
ENSMUSG00000029368	Aldob	Albumin	ENSMUSG00000066153	Mup21	Major urinary protein 21
ENSMUSG00000029445	Alb	4-hydroxyphenylpyruvic acid dioxygenase	ENSMUSG00000066154	Mup3	Major urinary protein 3
ENSMUSG00000030711	Hpd	Sulfotransferase family 1A, phenol-preferring, member 1	ENSMUSG00000068086	Cyp2d9	Cytochrome P450, family 2, subfamily d, polypeptide 9
ENSMUSG00000030895	Sult1a1	Hemopexin	ENSMUSG00000069922	Ces3a	Carboxylesterase 3A
ENSMUSG00000031722	Hpx	Haptoglobin	ENSMUSG00000073830	Mup14	Major urinary protein 14
ENSMUSG00000031762	Hp	Metallothionein 2	ENSMUSG00000073834	Mup11	Major urinary protein 11
ENSMUSG00000031765	Mt2	Metallothionein 1	ENSMUSG00000073842	Mup7	Major urinary protein 7
ENSMUSG00000032079	Mt1	Apolipoprotein A-V	ENSMUSG00000074373	NA(NA4)	Predicted gene 10680
ENSMUSG00000032081	Apoa5	Apolipoprotein A-III	ENSMUSG00000075391	NA(NA5)	Glyoxalase 1, pseudogene
ENSMUSG00000032083	Apoa4	Apolipoprotein A-I	ENSMUSG00000076258	NA(NA6)	Predicted gene, 23935
ENSMUSG00000033634	Apoc3	N-acetyltransferase 8 family member 2	ENSMUSG00000078672	Mup20	Major urinary protein 20
ENSMUSG00000033831	Apoa1	Fibrinogen beta chain	ENSMUSG00000078675	Mup16	Major urinary protein 16
ENSMUSG00000033860	Nat8f2 (Cml2)	Fibrinogen gamma chain	ENSMUSG00000078680	Mup10	Major urinary protein 10
ENSMUSG00000034957	Fgb	CCAAT/enhancer binding protein, alpha	ENSMUSG00000078686	Mup98	Major urinary protein 98
ENSMUSG00000034990	Fgg	Retinol binding protein 4	ENSMUSG00000079015	Serpina1c	Serine peptidase inhibitor, clade A, member 1C
ENSMUSG00000034994	Cebpa	Eukaryotic translation elongation factor 2	ENSMUSG00000080058	NA(NA7)	Predicted gene 11175
ENSMUSG00000035686	Eef2	Thyroid hormone responsive	ENSMUSG00000080374	NA(NA8)	Predicted gene 22953
ENSMUSG00000037071	Thrsp	Stearoyl-Coenzyme A desaturase 1	ENSMUSG00000083773	NA(NA9)	Predicted gene 13394
ENSMUSG00000037080	Scd1	Apolipoprotein A-IV	ENSMUSG00000083840	NA(NA10)	NI
ENSMUSG00000037095	Lrg1	Leucine-rich alpha-2- glycoprotein 1	ENSMUSG00000085834	NA(NA11)	Predicted gene 15622
ENSMUSG00000037798	Mat1a	Methionine adenosyltransferase 1A	ENSMUSG00000086432	NA(NA12)	RIKEN cDNA B430119L08 gene
ENSMUSG00000046687	Gm5424	Predicted gene 5424	ENSMUSG00000089873	Mup13	Major urinary protein 13
ENSMUSG00000047631	Apof	Apolipoprotein F	ENSMUSG00000091957	NA(NA13)	Ribosomal protein S2, pseudogene 10
ENSMUSG00000048087	Gm4737	Predicted gene 4737	ENSMUSG00000094793	Mup12	Major urinary protein 12
ENSMUSG00000049091	Sephs2	Selenophosphate synthetase 2	ENSMUSG00000096688	Mup17	Major urinary protein 17
ENSMUSG00000049422	Chchd10	Coiled-coil-helix-coiled-coil-helix domain containing 10	ENSMUSG00000097554	NA(NA14)	NI
ENSMUSG00000050445	Cyp8b1	Cytochrome P450, family 8 , subfamily b, polypeptide 1	ENSMUSG00000098178	NA(NA15)	NI
ENSMUSG00000050708	Ftl1	Ferritin light polypeptide 1	ENSMUSG00000098816	NA(NA16)	Predicted gene, 27786
ENSMUSG00000052974	Cyp2f2	Cytochrome P450, family 2, subfamily f, polypeptide 2	ENSMUSG00000098867	NA(NA17)	NI
ENSMUSG00000054422	Fabp1	Fatty acid binding protein 1	ENSMUSG00000099143	NA(NA18)	NI
ENSMUSG00000056501	Cebpb	CCAAT/enhancer binding protein			

Abbreviations: NI, no information

Supplementary table 2. Clinical data of patients with chronic hepatitis C (liver tissue analysis)

	Patients (Gender)	Age	ALT (U/l)	Alb (g/dl)	AFP (ng/ml)
HCC (-)	234 (F:126, M:108)	61.6±5.8	59.8±50.8	4.3±0.3	5.2±11.6
HCC (+)	33 (F:9, M:24)	65.3±5.4	79.4±44.7	4.0±0.4	51.9±136.2

The age and Alb value were significantly different between the two groups
($p<0.05$ student t-test).

Supplementary table 3. Clinical data of chronic hepatitis C patients (exosome analysis)

	Patients (Gender)	Age	ALT (U/l)	Alb (g/dl)	AFP (ng/ml)
HCC (-)	55 (F:29, M:26)	66.2±10.5	57.1±31.9	3.6±0.6	34.2±63.3
HCC (+)	15 (F:8, M:7)	71.8±8.1	54.5±25.2	3.8±0.5	18.6±19.2

No statistically significant difference was observed between all groups.

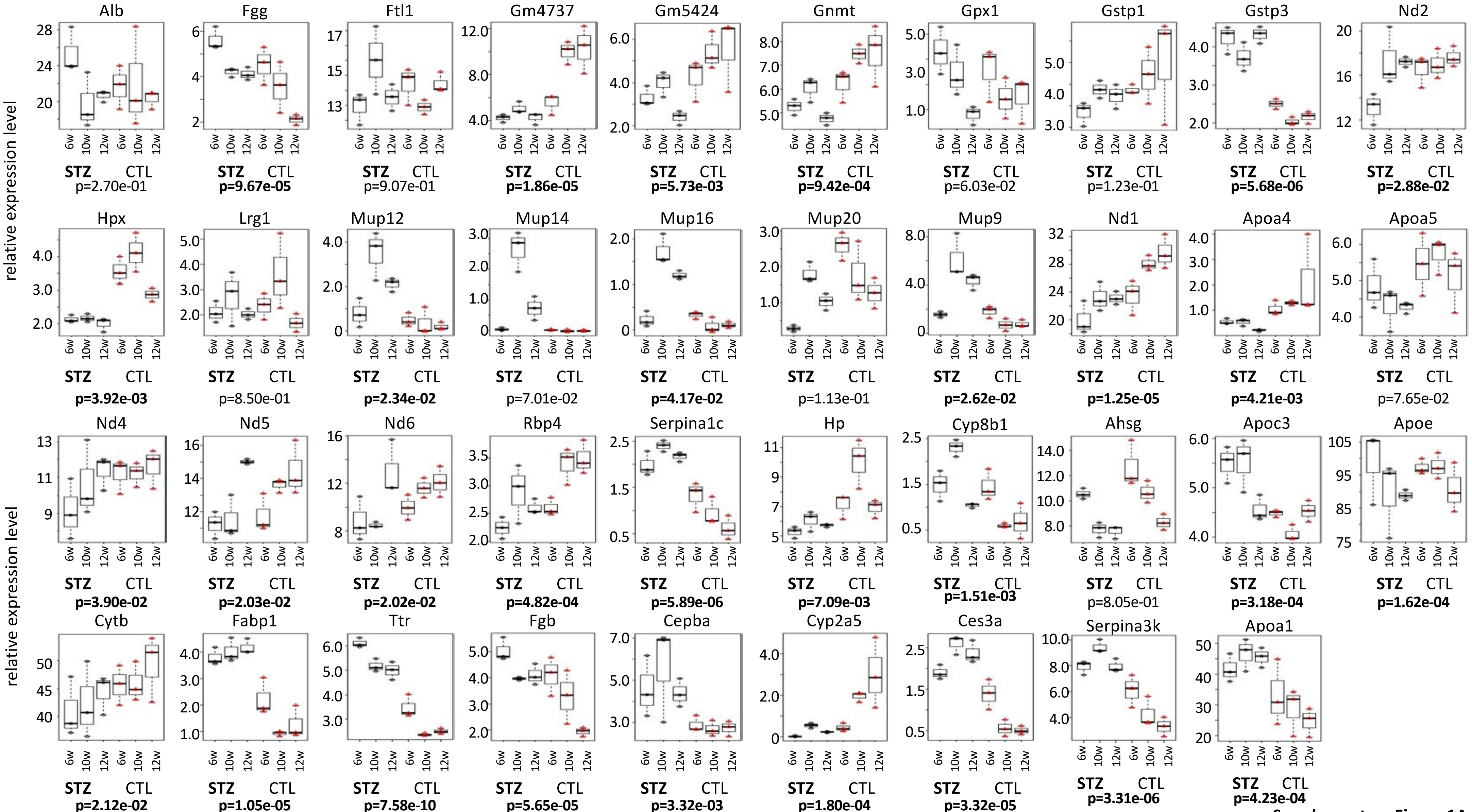

Supplementary Figure 1A

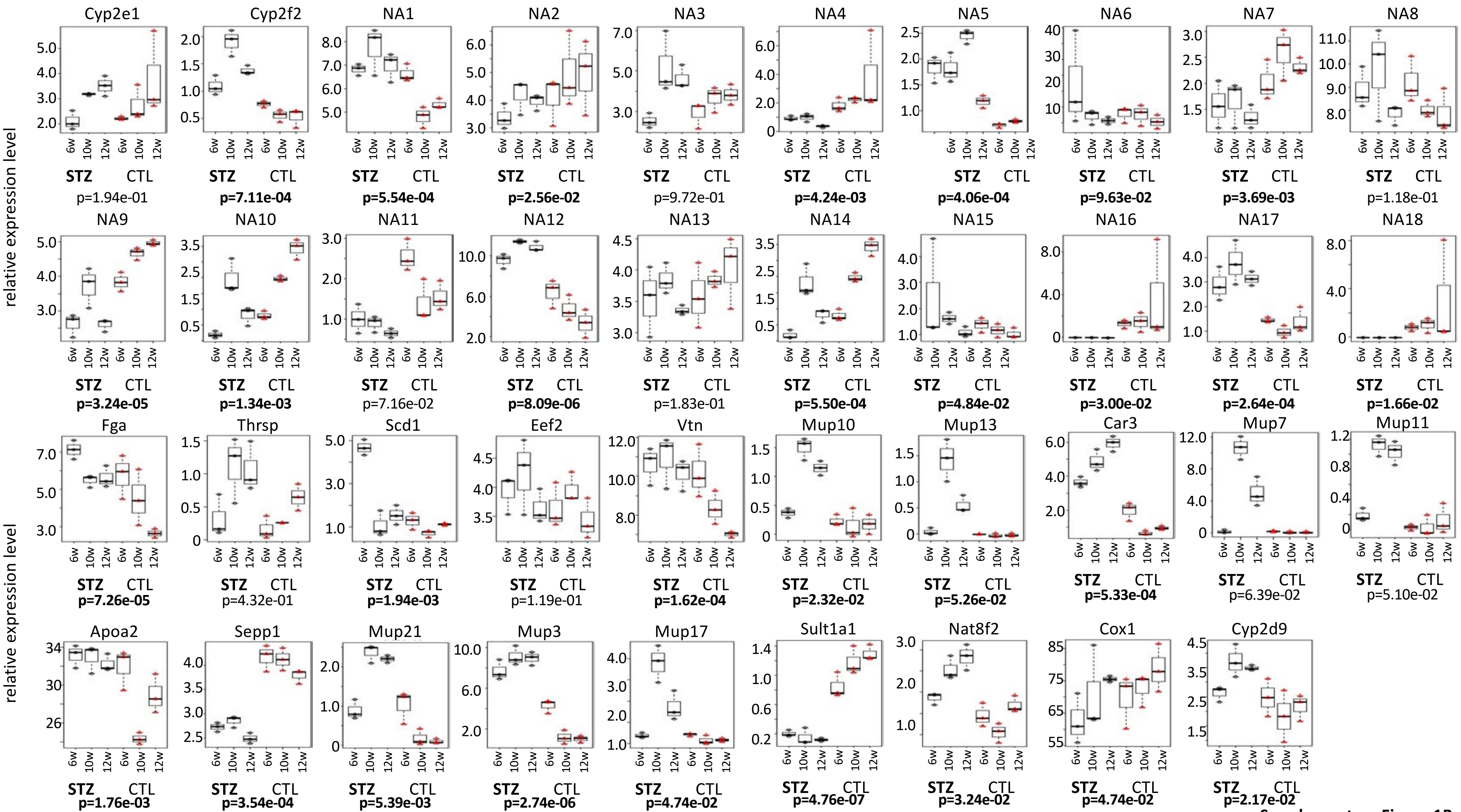

Supplementary Figure 1B
